# Vitamin A Status Improvement in Obesity: Findings and Perspectives Using Encapsulation Techniques

**DOI:** 10.3390/nu13061921

**Published:** 2021-06-03

**Authors:** Camila de Carvalho Gomes, Thais Souza Passos, Ana Heloneida Araújo Morais

**Affiliations:** 1 Postgraduate Program in Biochemistry and Molecular Biology, Center for Biosciences, Federal University of Rio Grande do Norte, Natal 59078 970, Brazil; camila.carvalhog@hotmail.com; 2 Department of Nutrition, Health Sciences Center, Federal University of Rio Grande do Norte, Natal 59078 970, Brazil; thais_spassos@yahoo.com.br; 3 Postgraduate Program in Nutrition, Health Sciences Center, Federal University of Rio Grande do Norte, Natal 59078 970, Brazil

**Keywords:** retinoic acid, adipose tissue, provitamin A, carotenoids, solubility, functionality

## Abstract

The association between obesity and vitamin A has been studied. Some studies point to the anti-obesity activity related to this vitamin, carotenoids with provitamin A activity, and carotenoid conversion products. This performance has been evaluated in respect of adipogenesis, metabolic activity, oxidation processes, secretory function, and oxidative stress modulation, showing a new property attributed to vitamin A in preventing and treating obesity. However, vitamin A and its precursors are highly sensitive and easily degraded when subjected to heat, the presence of light, and oxygen, in addition to losses related to the processes of digestion and absorption. In this context, encapsulation presents itself as an alternative capable of increasing vitamin A’s stability in the face of unfavorable conditions in the environment, which can reduce its functionality. Considering that vitamin A’s status shows a strong correlation with obesity and is an innovative theme, this article addresses the associations between vitamin A’s consumption and its precursors, encapsulated or not, and its physiological effects on obesity. The present narrative review points out those recent studies that demonstrate that vitamin A and its encapsulated precursors have the most preserved functionality, which guarantees better effects on obesity therapy.

## 1. Introduction

Studies point out that vitamin A is essential for maintaining human health, having a prominent role in developing the visual system and epithelial tissue. Retinoic acid (RA) is a metabolite responsible for most of the biological activities of vitamin A. Furthermore, it is known that this vitamin and retinoic acid contribute to normal metabolism, resistance to infection, and increased immunity [1]. Recently, vitamin A has stood out for its benefits in preventing metabolic diseases [2].

It is still necessary to intensify investigations related to the metabolism, assimilation, and bioavailability of vitamin A, especially considering the relationship with metabolic diseases. Bonet et al. [3] mentioned that bioavailability and vitamin metabolism are being studied in more depth, and with this, they are beginning to be understood. Thus, it has been suggested that vitamin A and carotenoids with provitamin A activity, offered within safe ranges, depending on individual genetic variants, adipose phenotype, body mass index (BMI), and body fat distribution, can help control obesity and improve metabolism.

In addition, Stenzel et al. [4] pointed out that vitamin A’s involvement in adiposity has been highlighted. Thus, the aid in adipose tissue metabolic regulation has been more evidenced. Saeed et al. [5] described that reduced serum retinol levels are related to obesity and other conditions, such as glucose intolerance, insulin resistance, hepatic steatosis, and hypertriglyceridemia.

According to Bento et al. [6], there is evidence that vitamin A is involved in body fat regulation, suggesting that when it is deficient, there is an increase in the recruitment of pre-adipocytes to adipocytes, apoptosis inhibition, and increased adaptive thermogenesis. Some studies also highlight the relationship between high BMI and low vitamin A concentrations, pointing out that hypovitaminosis may affect increasing obesity. In addition, it can regulate body weight through the action of the metabolites of this nutrient [7].

However, the use of vitamin A and its precursors present in food and the addition of these fat-soluble compounds in industrialized products are directly affected by low water solubility and oxidation. In this context, encapsulation techniques have offered possible solutions to increase bioavailability, water solubility, and vitamin A and carotenoid stability. Thus, sensitive ingredients are packed in a coating or wall material. Different methods for encapsulating carotenoids can be used, such as nanoemulsions, microemulsions, liposomes, solid lipid nanoparticles, and complex assemblies with macromolecules [8]. Nanoemulsions are presented as excellent options for protecting vitamin A and its precursors through encapsulation since they contain lipids, in which bioactive substances can be dissolved. They are also relatively transparent and can be incorporated into various food and beverage products [9].

Considering the importance of discussing the relationship between vitamin A and obesity, this article proposes to approach, through a literature review in a narrative and systematically organized way, the associations between the consumption of vitamin A and its encapsulated precursors or not, and associate it with its physiological effects in in vivo models with obesity. Descriptors and related terms were inserted in databases for the survey, selecting those involving vitamin A or its active metabolites, encapsulated or not. In addition, the performance and relationship between vitamin A and obesity were studied.

## 2. Vitamin A

Vitamin A is an essential micronutrient that cannot be synthesized by the human body, so it needs to be acquired through diet. It is present in various foods and is well distributed in nature, standing out in sources such as dairy products, fruits (yellow and orange colors), vegetables (dark green color), eggs, liver, and others [10].

In addition, vitamin A is a crucial component of promoting many biological functions, including reproduction, embryological development, cell differentiation, growth, immunity, and vision [11]. However, most bioactive properties are not performed by vitamin A but by its metabolites [12].

Vitamin A designates a family of hydrophobic compounds that include retinoids and carotenoids with provitamin A activity, meaning that there are several forms of this vitamin [13]. The main biologically active forms of vitamin A are retinol, retinal, and retinoic acid [14]. Retinoids include all-natural and synthetic compounds that structurally resemble vitamin A, including derivatives that are not biologically active [11,13].

β-carotene, α-carotene, and β-cryptoxanthin are considered the main precursors of vitamin A, highlighting β-carotene as the most potent. The metabolic conversion of β-carotene is chemically possible and is more efficient due to its molecular structure, which contains two unsubstituted β-ionone rings linked to the polyenic side chain (rich in conjugated double bonds). Thus, carotenoids can theoretically generate two molecules of vitamin A [15].

However, it is susceptible to oxidation by exposure to oxygen, light, and heat, resulting in loss of color, reduced antioxidant activity, and other biological activities [16]. In human nutrition, β-carotene contributes more than 30% to vitamin A intake in western countries and, in many populations, is the only source of vitamin A [17].

The conditions to assess vitamin A status are related to ensuring sufficient hepatic reserves (>20 μg/g liver) expressed in retinol activity equivalent. Thus, it is known that one RAE is identified as the biological activity attributed to 1 μg of all-trans-retinol, being influenced by the estimated effectiveness of absorption and conversion to retinol. In this context, it is equivalent to 24 μg of α-carotene or β-cryptoxanthin (which has one beta-ionone ring), and 12 μg of β-carotene (which has two beta-ionone-type rings). However, lycopene and xanthophylls such as lutein and zeaxanthin are not precursors of the vitamin in question [12]. Based on this, RAE expresses the vitamin A present in foods, and in some studies, this micronutrient is expressed in international units (IU). However, in recent studies, researchers have used milligrams (mg) to inform the amounts of this vitamin [10].

## 3. Vitamin A Metabolism

The gastrointestinal tract (GIT) consists of a series of barriers that precede the arrival of digestible products that can be absorbed into the capillaries in the subepithelial tissue. The main obstacles are the gastric and intestinal environment, mucus, narrow junctions that block the paracellular passage, the GIT epithelial cells, and the subepithelial tissue [18].

Reboul [19] emphasizes that the bioaccessibility of carotenoids, for example, is highly variable. This process is characterized by the carotenoid portion released from food and incorporated in micelles, capable of being absorbed by the intestine.

In a complementary way, Maurya et al. [20] describe that several factors can delay or inhibit the arrival of vitamins to the systemic circulation in an active form once ingested. In this sense, bioavailability is characterized and defined as the vitamin consumed that finally reaches the systemic circulation as an active form. There are factors such as chemical instability during the digestion process, low solubility in GIT fluids, slow absorption, and first-pass metabolism as mechanisms that directly impact these nutrients’ absorption.

In the gastrointestinal tract, carotenoids follow the same path as lipids. In the first part of digestion, dissolution occurs in the lipid phase of the meal, which is then emulsified in the stomach and duodenum [19]. Carotenoids with provitamin A activity need to be converted to retinol through oxidative cleavage performed by β-carotene monooxygenase present in the intestinal tract and absorbed by the intestine brush border cells [21].

A considerable part of free carotenoids and retinyl esters from the breakdown of carotenoids with provitamin A activity are involved in chylomicrons during the postprandial period and secreted in the lymph to reach the bloodstream [19]. Lintig et al. [22] describe that carotenoids can also be delivered to peripheral cells and captured in a process mediated by lipoprotein lipase. The remaining carotenoids in the chylomicrons are transported to the liver, incorporated into very-low-density lipoproteins (VLDLs), converted into low-density lipoproteins (LDLs) in the circulation, and are absorbed by LDL receptor-mediated endocytosis in peripheral cells.

Blaner et al. [23] report that dietary retinol (Vitamin A) is directly absorbed by the enterocytes that line the proximal small intestine. The dietary retinyl esters, however, cannot be absorbed intact by the intestinal mucosa. They suffer the action of the luminal retinyl ester hydrolase (REH) to produce free retinol.

It is known that post-digestion, vitamin A is incorporated into chylomicrons by intestinal cells. In the duodenum, lipoprotein lipase acts on chylomicrons, releasing the content that is absorbed by the small intestine [10]. Within the enterocyte, retinol is transferred bound to the type II cellular retinol-binding protein (CRBP II) to lecithin retinol acyltransferase (LRAT) responsible for further esterification. Then, the chylomicrons incorporate the retinyl ester and carry it to the lymphatic circulation, culminating in 20 to 60% of the total retinol efflux from the intestinal cells [21].

It then circulates from the plasma to the liver, in which approximately 70% of vitamin in excess is stored as retinyl ester molecules in hepatic stellate cells [10]. The rest is released into the circulation as retinol bound to retinol-binding protein 4 (RBP4) for transport to extrahepatic tissues. Retinyl ester molecules can also be carried to extrahepatic sites postprandially independent of RBP4. According to the literature, vitamin A stocks have already been observed in the pancreas, spleen, kidneys, larynx, and lung stellate cells [24].

Saeed et al. [25] emphasized that the liver plays a central role in the metabolism of vitamin A, as it produces the bile that participates in aiding intestinal absorption of fat-soluble nutrients, and produces RBP4 that distributes vitamin A, such as retinol, to peripheral tissues.

Vitamin A circulates in the blood bound to the serum retinol-binding protein (RBP) transported to the cells through a membrane protein stimulated by retinoic acid 6 (STRA6). Serum RBP levels have been reported to be elevated in rodents and humans with obesity, causing insulin resistance. The discovery suggests a mechanism that allows RBP to perform such an effect that STRA6 is a vitamin A transporter and has functioned as a surface signaling receptor [26].

When the liver’s vitamin A stores are depleted, RBP is retained in the hepatocyte endoplasmic reticulum and is not secreted. That is, in vitamin A deficiency, as with serum retinol, RBP is reduced. Due to the known ratio of retinol to RBP (1:1 ratio), the serum concentration of RBP reflects the serum concentration of retinol [27].

Plasma retinol concentrations are generally evaluated to monitor vitamin A requirements. However, their value is limited due to plasma retinol levels regulated by hepatic homeostatic control. In this way, they only decrease when the concentration of the vitamin in the liver is almost depleted [12].

Considering the serum concentrations of vitamin A, Timoneda et al. [12] state that a value below a cut-off point of 0.70 μmol / L of retinol represents a condition of vitamin A deficiency. Thus, this vitamin’s concentrations are sufficiently low to increase the risk of adverse effects. Therefore, these individuals are more susceptible to infections and have higher mortality rates than those with adequate vitamin A status [10].

In addition, as vitamin A plays a role in preventing metabolic diseases, it has been proposed to affect obesity and diseases related to this condition. Thus, retinoic acid has been identified as responsible for many reported associations [2].

## 4. Association between Obesity, Vitamin A, and Carotenoids (Provitamin A Activity)

Scientific evidence has shown that in the nutritional status of obesity, a decrease in liposoluble vitamin concentrations in the plasma could occur because of lower ingestion of these micronutrients and/or high storage in the adipose tissue. Because these vitamins are fat-soluble, they are stored in adipocytes, which decrease body bioavailability [28].

Trasino et al. [29] observed that obesity causes a “silent” vitamin A deficiency marked by reductions in this nutrient’s liver levels and signaling in multiple organs. It was demonstrated for the first time that even with adequate vitamin A in the diet, in obesity, there is a drastic reduction in vitamin concentration.

**Figure 1:**
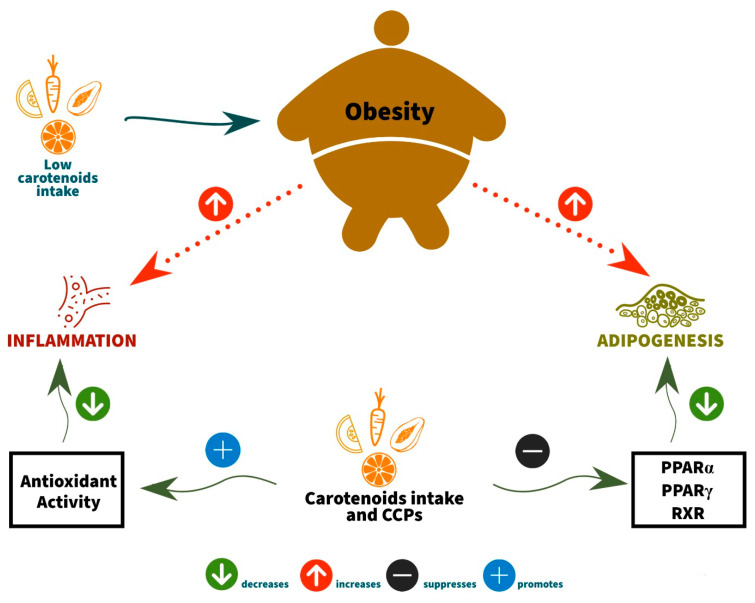
Relationship between carotenoids (β-carotene, α-carotene, and β-cryptoxanthin) or their conversion products and obesity. CCPs: carotenoid converting products. PPARγ: peroxisome proliferator-activated receptor gamma. PPARα: peroxisome proliferator-activated receptor alpha. RXR: retinoid X receptor.

According to Coronel et al. [30], vitamin A, similar to the hormones with pleiotropic effects in mammals, is an important adipose tissue development regulator and plays an essential role in obesity. These researchers described that individuals with obesity had reduced plasma β-carotene concentrations through the data obtained compared to eutrophic individuals. In addition, inflammation caused by oxidative stress is present in the nutritional status of obesity. Consequently, adipocyte differentiation and oxidative stress are targets for carotenoid action (Figure 1).

### 4.1. The Anti-Obesity Activities of Carotenoids and Carotenoid Converting Products (CCPs) by Inhibition of Adipocyte Differentiation

The anti-obesity actions of carotenoids and CCPs have been observed in preclinical studies. The mechanisms involved have begun to be revealed, suggesting that these compounds may help prevent and control obesity. In these studies, carotenoid and CCP anti-obesity action have been attributed to several tissue effects, mainly adipose tissue [3,31,32].

Most of the effects occur through adipocyte differentiation inhibition. Thus, this is linked to the decrease in the expression of peroxisome proliferator-activated receptor gamma (PPARγ) in the adipose tissue and decreased retinoid X receptor (RXR) expression. β-carotene inhibits adipogenesis by the β-apo-140-carotenal production and suppression of PPARα, PPARγ, and RXR activation, in addition to the all-trans retinoic acid output. In addition, the xanthophyll β-cryptoxanthin suppresses adipogenesis via retinoic acid receptor (RAR) activation [32].

The possible carotenoid protective effect on insulin resistance may result from the α- and β-carotene ability to serve as precursors of retinoic acid [33]. Retinoic acid, the nuclear receptor ligand of the RAR and RXR types, which forms a heterodimer with the γ receptor activated by a peroxisome proliferator and other nuclear receptors, can act in regulating glucose by increasing insulin sensitivity or insulin release [34].

### 4.2. The Anti-Obesity Activities of Carotenoids and Carotenoid Converting Products (CCPs) by Antioxidant Capacity

Through the antioxidant capacity of carotenoids, these compounds are also related to another protective mechanism for health. Oxidative stress and inflammation are marked changes that occur in the nutritional status of obesity. In this context, carotenoids have been identified as molecules that stand out due to their antioxidant and anti-inflammatory properties [35]. The reported associations between serum carotenoids, C-reactive protein (CRP), and adiponectin are consistent with previous findings, which reported an inverse relationship between plasma concentrations of carotenoids and interleukin-6 (IL-6), PCR, tumor necrosis factor-alpha (TNF-α), and retinol-binding protein 4 (RBP4).

The exact mechanisms that explain the carotenoids’ lower concentrations in the nutritional status of obesity still are unclear. Östh et al. [36] verified that the β-carotene concentrations in adipocytes isolated from the subcutaneous adipose tissue of obese individuals were 50% lower than β-carotene concentrations measured in normal or non-obese individuals. Harari et al. [33] proposed that, since obesity triggers high oxidative stress and inflammatory condition, it may induce the depletion of carotenoids and retinoic acid. Therefore, this can justify the low carotenoid concentrations found in the circulation and the adipose tissue.

Several studies describe the inverse relationship between obesity and vitamin A or β-carotene (precursor to vitamin A). Beydoun et al. [37] mention that serum levels of carotenoids, particularly β-carotene and retinyl esters, are inversely associated with metabolic syndrome (MS). Wei et al. [7] reported that serum vitamin A in school-age children in Chongqing (China) with obesity was significantly lower than the overweight and eutrophic groups, establishing a relationship between obesity and hypertriglyceridemia, hyperglycemia, and MS with vitamin A insufficiency.

Vitamin A deficiency induces stress in the endoplasmic reticulum, leading to pancreatic islet cell apoptosis, inhibits the activation of the insulin signaling cascade in insulin-sensitive tissues, and limits the hepatic glucokinase activity of hepatic glucose metabolism [38]. It has also been reported that vitamin A deficiency, as measured by low blood concentrations, may be associated with insulin resistance in adult humans with obesity [7]. Godala et al. [39] found that individuals with vitamin A deficiency have a higher concentration of glucose than individuals with metabolic syndrome with a normal vitamin A concentration. In the conclusion of their study, Stenzel et al. [4] emphasize that obesity has an inverse association with vitamin A status. This relationship is stronger when the individual with obesity presents another type of associated metabolic alteration.

Kuang et al. [40] observed that vitamin A status contributes to regulating hepatic glucose in lipid metabolism and can regulate carbohydrate metabolism. It can also interfere with weight gain and the development of obesity. However, Blaner [2] reported that, in general, β-carotene reduces obesity only when it is converted to vitamin A and mentioned that more than 90% of the total vitamin A present in the body is found in the liver. Mody [41] pointed out that this organ has a high concentration of retinyl esters, particularly retinyl palmitate, which is the main form of vitamin A storage. It is important to note that this accumulation will be linked to the vitamin’s nutritional status, with a change in concentration according to the adequate or insufficient intake of this micronutrient.

Coronel et al. [30] mentioned that studies on animal models showed that treatment with retinoic acid generates positive effects on adipogenesis, leading to the darkening of fatty tissue and inducing adipocyte fatty acid oxidation. These effects were similar in the liver and muscle when evaluated in animal models and cell cultures. Observational and intervention studies reinforce these findings and supply a mechanistic explanation for why fruit and vegetable intake, rich in carotenoids with provitamin A activity, prevent obesity.

Most of these findings support the beneficial effects of various carotenoids on obesity. They suggest that carotenoids may play an important role in adipose tissue, modifying multiple parameters related to obesity and/or associated comorbidities (Table 1).

## 5. Encapsulation Technology and Vitamin A

Bioactive compound encapsulation was developed to increase stability, apparent solubility, and industrial application, and to improve compound bioavailability [42]. It is a rapidly expanding technology in which droplets or particles of liquid or solid material are involved with a polymeric material, providing protection against adverse environmental conditions and controlling the release characteristics of coated materials (core).

Microencapsulation is characterized by particles with diameters ranging from 1 to 1000 micrometers [43]. According to Suganya and Anuradha [44], nanoencapsulation aims to incorporate a bioactive into an encapsulating agent in the nanoscale range. Thus, nanoencapsulation can increase bioavailability, improving the controlled release and accurately targeting the delivery of bioactive compounds to a greater extent than microencapsulation. Nanoparticles are materials whose dimensions range from 1 to 100 nanometers [45].

In recent years, nanoencapsulation technology has been a promising strategy in the food industry because of its advantages. Nanoencapsulation is generally used to provide different nutraceutical products composed of vitamins, antioxidants, and many other molecules and bioactive compounds, allowing for improved functionality and stability. It also protects bioactives and nutrient encapsulated assets from environmental, enzymatic, and chemical changes, and improves sensory attributes [17].

Vitamin A and carotenoids with provitamin A activity are excellent candidates to benefit from this technology, as it degrades quickly due to their sensitivity to heat, light, moisture, and oxygen [46]. Encapsulation also increases the solubility of lipophilic substances in aqueous media, in addition to slowing and/or preventing its degradation until the bioactive is delivered to the place target where absorption will occur [47] (Table 2).

Maurya et al. [20] describe that vitamin A encapsulation has attracted food technologists due to increasing the potential to incorporate in food matrices, improve bioavailability, and promote the controlled release without influencing the sensory quality of food matrices, preserving product acceptability.

However, encapsulation success depends on selecting an appropriate encapsulating agent and the encapsulation method. The coating type will determine the encapsulation efficiency, particle stability, and product characteristics [48]. Azeredo [49] described that the encapsulating material’s nature mainly influences the bioactive’s stability. Polysaccharides, proteins, and lipids are materials with the potential for encapsulating use. Standing out are zein (corn prolamine), whey proteins, gelatin, gums (gum arabic, alginate, guar gum, and others), cellulose (crystalline micro cellulose), starch, maltodextrins, and cyclodextrins. Vitamin A, intended for dietary purposes, is encapsulated mainly by gelatin produced from animal bones, skin, and tendons [46].

The encapsulation method is chosen based on the core material’s physical and chemical properties and the encapsulating agent, the particle’s desirable shape and diameter, and the desirable controlled release profile. The techniques used include coacervation (phase separation), spray drying and cooling, emulsion systems, solid lipid nanoparticles, liposomes, and an inclusion complex [48].

It is essential to understand the biological processes that regulate micronutrient absorption and bioavailability to design efficient nanoparticles containing vitamin A [20]. Therefore, vitamin A would benefit from a nanoencapsulation technique. It promotes water-solubility, linked to the reduction of particle size at the nanoscale; delay and/or avoidance of degradation processes; and promoting release at the target site to achieve the desired absorption [21].

The choice of encapsulating agent and technique will directly influence the incorporation efficiency. Sachaniya et al. [50] developed liposomes containing phosphatidylcholine and vitamin A produced by the lipid film hydration method followed by sonication and extrusion. Obtained results indicated that about 95% of free vitamin A was released in 24 h, while only 50% of vitamin A encapsulated was released. 

Liang et al. [51] produced nanoparticles by the oil-in-water method using modified starch in association with β-carotene. They observed its retention in nanoemulsions was significantly higher compared to the free and dispersed pigment in oil. After in vitro digestion, β-carotene bioaccessibility was increased from 3.1 to 35.6% through nanoencapsulation. Therefore, the data indicated that the micronutrient in the colloidal particles had a larger surface area and would benefit the lipase interaction.

Rocha et al. [52] also observed the benefits related to encapsulation. They demonstrated that the performance of β-carotene in aqueous media was highly superior when loaded in nanoparticles, which also allowed for more efficient biological activity. In addition, Baek et al. [53] reinforced that nanoparticles containing β-carotene showed more excellent stability, providing increased potential for application in the food industry.

Medeiros et al. [54] encapsulated the carotenoid extract from Cantaloupe melon, containing mainly beta-carotene, in porcine gelatin and whey protein isolate by emulsification O/A technique. In this way, the researchers evaluated which encapsulating agent could enable water dispersibility and carotenoid stability in yogurt. The results showed that gelatin nanoencapsulation significantly increased water-solubility and color stability in yogurt over the 60 days of shelf life. According to the authors, the observed benefits were related to the particle size obtained of approximately 60 nm, which increased the contact surface with water and enhanced the carotenoid functionality.

Oliveira et al. [55] evaluated the antioxidant potential and stability of the same extract rich in nanoencapsulated carotenoids (CE) from cantaloupe melon. The results showed that nanoencapsulation potentiated the antioxidant activity by approximately 60% due to the complex chemical interactions between porcine gelatin and crude carotenoid extract. As a result, more antioxidant potential preservation of carotenoids was observed in storage conditions for 60 days at room temperature in the light (46.87 (2.25)%) and dark (58.5 (1.94)%), and under refrigeration in the light (1600 lux) (49.59 (2.25)%) and dark (68.7 (4.19)%), when compared to non-encapsulated carotenoids.

O/W nanoemulsions remain the most widely used fat-soluble bioactive encapsulation systems in the food industry. In the studies by [50,51,54], who evaluated the positive effects of nanoencapsulation under the action and protection of bioactive compounds, it was observed that the proportions of surfactants used and the interactions produced in the system strongly influence the functional properties and physical and chemical stability of the particles obtained. Among the surfactants most used to promote vitamin A and β-carotene nanoencapsulation, whey protein isolate, casein, soluble soy polysaccharides, spirulina peptides, egg phosphatidylcholine, polysaccharides modified with octenyl anhydride succinic, lecithin, saponins, and β-lactoglobulin have been highlighted [9].

## 6. Prospects for Vitamin A Encapsulated in Food

Adding bioactive compounds to food products is a technological challenge. The main difficulty in using these components is the high susceptibility to a complex food matrix and the gastrointestinal tract (temperature, light, oxygen, pH, enzymes, and others). Although researchers strive to successfully translate drug encapsulation technology to delivery bioactives to protect them during production, storage, transportation, and consumption, success is still limited. This fact is associated with the need to adopt technological processes that guarantee security, acceptability, and industrial production [58].

Recent oral therapy trends based on nanoparticles have intensified studies to increase the solubility, permeability, and stability of micronutrients and bioactives. One of the current discussions relates to achieving high bioavailability of compounds with low absorption or instability [59]. Lundquist and Artursson [18] reinforced that nanoparticles can be used as a tool capable of overcoming physiological and biochemical barriers to the absorption of a micronutrient or bioactive compound in the gastrointestinal tract.

It is important to remember that β-carotene also presents as a source of provitamin A and is also a natural pigment. However, the use is still limited due to its instability and low bioavailability [60]. Therefore, encapsulation can be used to improve its dispersibility in water and chemical stability in food [56,61], in addition to enhancing therapeutic effects [62].

In addition, it can improve bioavailability with nanomaterials that can facilitate metabolism in the liver [63] and encapsulating agents that can assist in the vitamin’s paracellular transport through narrow cell junctions [20]. Increased water-dispersibility and stability of vitamin A can be promoted by incorporation into encapsulating agents with beneficial physical and chemical properties by appropriate encapsulation techniques [48].

The small nanoemulsion droplet sizes can increase the encapsulated hydrophobic nutraceutical bioavailability due to the faster and more complete lipid phase digestion [64]. Liu et al. [56] observed that cellular absorption of nanoparticles contributed to the higher concentration of β-carotene in cells, and that β-carotene is not bioaccessible until it is incorporated into micelles in the intestine. After encapsulation in nanoparticles, β-carotene can be dispersed in water and absorbed in the intestine.

Resende et al. [13], in in vitro tests simulating gastrointestinal digestion, pointed out that the nanoparticles are not altered in the stomach and that the formulations’ biocompatibility did not show fibroblast toxicity. By administering nanoparticles, 80% of vitamin A reached the intestine in the digestibility test, successfully promoting nanotechnology’s beneficial human health effects.

Liu et al. [57] showed that β-carotene encapsulation could significantly improve this compound’s storage stability. The bioactive compound loaded in two types of liposomal systems is released slowly in the stomach, and more than 70% is released in the intestine. The results showed that liposomes could promote the protection of active substances against damage in the stomach and release them in the small intestine, where they will be absorbed. Thus, it is perceived that the encapsulation of vitamin A or its precursors brings essential benefits for these components for better use by the body and consequently metabolic effects that favor the obesity treatment.

Therefore, vitamin A and its precursors affect adipogenesis, lipid catabolism, bio-synthesis, and the release of bioactive factors. However, it is unclear and there is no consensus as to whether vitamin A precursors, such as β-carotene, reduce the common factors in obesity only when converted. However, vitamin A and its precursors are easily degraded. Thus, studies have shown that the encapsulation techniques help stabilize, enable water dispersibility, improve the digestion, absorption, and potentialize the functionality of these nutrients in the body (Table 2).

## 7. Conclusions

The present narrative review has pointed out those recent studies that demonstrate that vitamin A and its encapsulated precursors have the most preserved functionality, which guarantees better effects on obesity therapy.

In this context, recognizing the instability of vitamin A and carotenoids with provitamin A activity in the face of environmental conditions, and thereby incorporating these bioactive compounds in encapsulating agents that bring stability and promote positive absorption effects, is a strategy of considerable interest. In this way, it is possible to expand this nutrient’s use in food or pharmaceutical products, as well as potentiating the effects in the treatment of deficiencies related to vitamin A.

Thus, it is believed that the encapsulation of vitamin A or its precursors brings essential benefits since it preserves and maintains functionality. It also favors these compounds’ action in target tissues and/or organs, thus allowing these bioactive compounds to be better used in the treatment of obesity and associated comorbidities.

Finally, it is worth highlighting the need for further studies to clarify the effects of vitamin A on preventing and controlling obesity through several known mechanisms related to free and encapsulated forms.

## Figures and Tables

**Table 1:** Scientific findings related to vitamin A and precursor status and obesity.

Reference	Study Type	Objectives	Findings Related to Obesity
Östh et al. (2014) [36]	Clinical	Evaluate clinical parameters such as triglycerides, total cholesterol, HDL/LDL, fasting glucose and insulin, β-carotene concentration in isolated subcutaneous abdominal adipocytes obtained from eutrophic, overweight, obese, and obese humans with type 2 diabetes.	It was observed that the concentration of β-carotene was 50% lower in the adipocytes of obese and obese diabetic groups in comparison with the eutrophic and overweight groups. Thus, the concentration of β-carotene in adipocytes appears to be inversely related to the nutritional status of obesity.
Trasino et al. (2015) [29]	Ex-vivo and pre-clinical	Analyze the histology of frozen human liver and mice to demonstrate that even with adequate vitamin A in the diet, obesity dramatically reduces vitamin A levels and signaling in major organs.	Obese mice (induced by eating a high-fat diet or genetic mutations) drastically reduced retinol levels in various organs. Organs from obese rats show impaired vitamin A transcriptional signaling, including reductions in retinoic acid receptor mRNAs and lower levels of intracellular retinol-binding protein Crbp1 (RBP1) in vitamin storage in stellate cells. Reductions in vitamin A signaling in the organs of obese mice correlate with increased adiposity and fatty liver.
Wei et al. (2016) [7]	Cross-sectional	Examine the association of vitamin A status with obesity and metabolic syndrome (MS) in school-aged children, assessing body height, weight, waist circumference, blood pressure, blood glucose, and lipids.	The serum vitamin A levels in the obese group were significantly lower than the overweight and eutrophic group (*p* < 0.05). BMI, waist circumference, and fasting glucose were significantly higher in the group with low vitamin A concentration (*p* < 0.05). As a result, vitamin A deficiency was significantly associated with metabolic syndrome (*p* < 0.05).
Godala et al. (2017) [39]	Case-control study	Assess the risk of vitamin A, C, E, and D deficiency in the plasma of patients with multiple sclerosis.	The results showed that plasma vitamin A levels were significantly lower (*p* < 0.05) in patients with metabolic syndrome. They also showed that hyperglycemia increased the risk of vitamin A deficiency.
Stenzel et al. (2018) [4]	Observational	Investigate the relationship between the state of antioxidant micronutrients and the components of metabolic syndrome in metabolically healthy (MH) and unhealthy (MU) obesity.	The results showed that obese MU adolescents have a significant inverse association between vitamin A and waist circumference.
Harari et al. (2020) [33]	Cohort	Evaluate the relationships between adipose tissue and serum carotenoids with body fat, abdominal fat distribution, muscle insulin resistance, adipose and liver tissue, and food intake. In addition, evaluate the relationships and distributions of carotenoids detected in adipose tissue and serum compared to serum carotenoids and retinol concentrations in individuals with and without obesity.	All serum carotenoid concentrations were significantly lower (*p* < 0.05) in samples collected from individuals with obesity compared with eutrophic individuals. Similar to serum carotenoids, most carotenoids in adipose tissue were inversely related to anthropometric measurements (weight, BMI, waist circumference, and total and central body fat). Total carotenoids, β-carotene, and α-carotene were inversely associated with fasting insulin and HOMA-IR. The insulin resistance of the adipose tissue correlated inversely with the total concentration of carotenoids.

**Table 2:** Encapsulation of vitamin A and precursors with provitamin A activity.

Reference	Objective	Encapsulation Technique	Materials	PD	EE (%)	Functionalities Associated	Related Mechanisms
Liu et al. (2019) [56]	Investigate the potential of particles in improving the absorption of active compounds such as β-carotene, using Caco-2 cells in vitro and small intestine ex vivo.	Emulsification oil in water (O/W)	β-carotene, barley protein, canola oil.	351 nm	90.7%	Uptake is dependent on time, concentration, and temperature. The uptake and transport of encapsulated β-carotene were higher (*p* < 0.05) (15%) compared to the free form (2.6%). There was also a greater capacity for retention and permeation in the intestinal tissue.	The particles could more easily enter Caco-2 cells through endocytosis. Therefore, the particle size is inversely proportional to the presence of β-carotene in the cell monolayers.
Rocha et al. (2018) [52]	Evaluate the effects of particles containing β-carotene on the activity of the enzymes glutathione-S-transferase (GST) and acetylcholinesterase (AChE) (Drosophila melanogaster homogenate), superoxide dismutase (SOD), and catalase and the cytotoxic properties in tumor cell lines and not tumoral.	Solid dispersion	Tween-80, β-carotene and polyvinylpyrrolidone (PVP).	337 nm	-	High dispersion in water and modulating AChE, presenting a high potential for controlling the cholinergic system. In low concentrations, particles showed mimetic activity in vitro for SOD and altered activity for GST. Particles also showed activity against four different tumor cell lines.	The performance of the carotenoid encapsulated in aqueous medium was significantly improved due to the reduction of the particle size, allowing enhancement of its biological activity.
Liu et al. (2020) [57]	Encapsulate vitamin C and β-carotene in liposomes (L-VC-βC) and evaluate structural characteristics, stability (4 °C and 25 °C), antioxidant activity, in vitro gastrointestinal digestion, and release kinetics.	Liposomes	Egg yolk lecithin, cholesterol, β-carotene, and vitamin C.	~250 nm	~98%	The storage stability of L-VC-βC was higher (*p* < 0.05) compared to L-βC. In gastrointestinal digestion, the carotenoid present in the two liposomes was released slowly, with more than 70% being released in the simulated gastric fluid.	In the gastric phase, the release of βC was proportional to the time of contact with the gastric fluid. In the intestinal phase, expanded, cracked, and fragmented liposomes were observed, possibly due to contact with bile salts, or due to increased membrane fluidity due to the permeation of bile salts, resulting in high adsorption of lipases.
Resende et al. (2020) [13]	Develop and characterize lipid particles containing vitamin A for food fortification, ensuring oral stability and bioaccessibility.	Organic solvent-free sonication method	Tween 80, vitamin A (VA), Gelucire (GEL), stearic acid (SA) and oleic acid (OA), Miglyol (MIG).	228 nm to 612 nm	5% to 97%	In vitro tests simulating gastrointestinal digestion showed that the particles were not altered in the stomach and that the biocompatibility of the formulations did not show toxicity in fibroblasts. With the developed particles, 80% of the added vitamin reached the intestine in the digestibility test.	After two hours, the size remained unchanged, showing stability of the particles in the stomach. In the intestine, bile salts, together with pancreatic lipases, can promote the digestion of the di- and triacylglycerols that make up the particles. Loss of structure and lipid aggregation can occur, as indicated by the increase in particle diameter.
Baek et al. (2020) [53]	Obtain nanoemulsions containing β-carotene (BC-NEs) and coated with water-soluble chitosan (WSC-BC-NEs) to improve the stability of β-carotene against high temperature and ultraviolet light.	Emulsification O/W	Medium chain triglyceride (MCT) oil, Tween 80, lecithin, β-carotene (BC), and chitosan.	BC-NEs = 64 nm. WSC-BC-NEs = 218 nm.	-	WSC-BC-NEs (2% chitosan) increased the thermal and ultraviolet stability of the encapsulated BC compared to BC-NEs. After 21 days of storage at 37 °C, WSC-BC-NEs preserved about 96.7% of β-carotene and 77.6% in UV light exposure (253 nm) at room temperature.	BC was dispersed in a lipid, which can be surrounded by additional protective layers. The increased stability can be explained by the limited interaction between the BC contained in the droplet nucleus and oxygen and radicals in the environment, due to the physical barrier provided by the wall material.
Medeiros et al. (2019) [54]	Produce nanoparticles based on carotenoids from cantaloupe melon pulp (*Cucumis melo* L.) rich in β-carotene (CE), and evaluate water dispersion and color stability in yogurt for 60 days of storage.	Emulsification O/W	CE, soybean oil, Tween 20, porcine gelatine (EPG), whey protein concentrate (EWPC), and isolated (EWPI).	EWPC = 123 nm EWPI = 161 nm EPG = 59.3 nm	EWPC = 77% EWPI = 58% EPG = 90%	EPG showed an increase in water solubility by about 267% compared to the crude extract. EPG added to yogurt simulated yellow fruit-flavored yogurt coloration and promoted high color stability and homogeneity compared to the CE.	The smallest particle size of EPG associated to the high chemical interaction between the CE and porcine gelatin justify the increase in water solubility and color stability.
Oliveira et al. (2021) [55]	Evaluate if the nanoencapsulation of the carotenoids of cantaloupe melon pulp (*Cucumis melo* L.) rich in β-carotene (CE) promoted the preservation or enhancement of the antioxidant potential when stored at different conditions (light and temperature).	Emulsification O/W	CE, soybean oil, Tween 20, porcine gelatin (EPG).	90.9 nm	94.80%	Carotenoid antioxidant activity increased after nanoencapsulation in porcine gelatin (57–59%). After 60 days, EPG preserved the β-carotene in the light (83.1%) and dark at 25 °C (99.0%) and in the dark (99.0%) at 5 °C, maintaining the antioxidant potential (68.7–48.3%) compared to CE.	The chemical interaction between carotenoid-protein may be related to the increase in water solubility. In addition, the photoprotective effects (absorption/dissipation of energy or extinction of reactive oxygen species) promote increased activity and antioxidant stability of carotenoids.

* PD: particle diameter. EE: encapsulation efficiency.

## References

[1] Xiao S., Li Q., Hu K., He Y., Ai Q., Hu L., Yu J. (2018). Vitamin A and Retinoic Acid Exhibit Protective Effects on Necrotizing Enterocolitis by Regulating Intestinal Flora and Enhancing the Intestinal Epithelial Barrier. Arch. Med. Res..

[2] Blaner W.S. (2019). Vitamin A signaling and homeostasis in obesity, diabetes, and metabolic disorders. Pharmacol. Ther..

[3] Bonet M.L., Ribot J., Galmés S., Serra F., Palou A. (2020). Carotenoids and carotenoid conversion products in adipose tissue biology and obesity: Pre-clinical and human studies. Biochim. Biophys. Acta Mol. Cell Biol. Lipids.

[4] Stenzel A.P., Carvalho R., Jesus P., Bull A., Pereira S., Saboya C., Ramalho A. (2018). Serum Antioxidant Associations with Metabolic Characteristics in Metabolically Healthy and Unhealthy Adolescents with Severe Obesity: An Observational Study. Nutrients.

[5] Saeed A., Hoogerland J.A., Wessel H., Heegsma J., Derks T.G.J., Van Der Veer E., Mithieux G., Rajas F., Oosterveer M.H., Faber K.N. (2020). Glycogen storage disease type 1a is associated with disturbed vitamin A metabolism and elevated serum retinol levels. Hum. Mol. Genet..

[6] Bento C., Matos A.C., Cordeiro A., Ramalho A. (2018). Vitamin A deficiency is associated with body mass index and body adiposity in women with recommended intake of vitamin A. Nutr. Hosp..

[7] Wei X., Peng R., Cao J., Kang Y., Qu P., Liu Y., Xiao X., Li T. (2016). Serum vitamin A status is associated with obesity and the metabolic syndrome among school-age children in Chongqing, China. Asia Pac. J. Clin. Nutr..

[8] Gul K., Tak A., Singh A.K., Singh P., Yousuf B., Wani A.A. (2015). Chemistry, encapsulation, and health benefits of β-carotene—A review. Cogent Food Agric..

[9] Banasaz S., Morozova K., Ferrentino G., Scampicchio M. (2020). Encapsulation of Lipid-Soluble Bioactives by Nanoemulsions. Molecules.

[10] Zinder R., Cooley R., Vlad L.G., Molnar J.A. (2019). Vitamin A and Wound. Nutr. Clin. Pract..

[11] Polcz M.E., Barbul A. (2019). The Role of Vitamin A in Wound Healing. Nutr. Clin. Pract..

[12] Timoneda J., Rodríguez-Fernández L., Zaragozá R., Marín M.P., Cabezuelo M.T., Torres L., Viña J.R., Barber T. (2018). Vitamin A deficiency and the lung. Nutrients.

[13] Resende D., Lima S.A.C., Reis S. (2020). Nanoencapsulation approaches for oral delivery of vitamin A. Colloids Surf. B Biointerfaces.

[14] Dattola A., Silvestri M., Bennardo L., Passante M., Scali E., Patruno C., Nisticò S.P. (2020). Role of Vitamins in Skin Health: A Systematic Review. Curr. Nutr. Rep..

[15] Amaya D.B.R. (1997). Carotenoids and Food Preparation: The Retention of Provitamin A Carotenoids in Prepared, Processed, and Stored Foods.

[16] Corrêa-Filho L.C., Lourenço M.M., Moldão-Martins M., Alves V.D. (2019). Microencapsulation of β-Carotene by Spray Drying: Effect of Wall Material Concentration and Drying Inlet Temperature. Int. J. Food Sci..

[17] Battistoni M., Bacchetta R., Di Renzo F., Metruccio F., Menegola E. (2020). Effect of nano-encapsulation of β-carotene on Xenopus laevis embryos development (FETAX). Toxicol. Rep..

[18] Lundquist P., Artursson P. (2016). Oral absorption of peptides and nanoparticles across the human intestine: Opportunities, limitations and studies in human tissues. Adv. Drug Deliv. Rev..

[19] Reboul E. (2019). Mechanisms of Carotenoid Intestinal Absorption: Where Do We Stand?. Nutrients.

[20] Maurya V.K., Aggarwal M., Ranjan V., Gothandam K.M. (2020). Improving Bioavailability of Vitamin A in Food by Encapsulation: An Update. Nanosci. Med..

[21] Sauvant P., Cansell M., Sassi A.H., Atgié C. (2012). Vitamin A enrichment: Caution with encapsulation strategies used for food applications. Food Res. Int..

[22] von Lintig J., Moon J., Lee J., Ramkumar S. (2020). Carotenoid metabolism at the intestinal barrier. Biochim. Biophys. Acta Mol. Cell Biol. Lipids.

[23] Blaner W.S., Li Y., Brun P.J., Yuen J.J., Lee S.A., Clugston R.D. (2016). Vitamin A Absorption, Storage and Mobilization.

[24] Nishimoto K., Toya Y., Davis C.R., Tanumihardjo S.A., Welham N.V. (2020). Dynamics of vitamin A uptake, storage, and utilization in vocal fold mucosa. Mol. Metab..

[25] Saeed A., Dullaart R.P.F., Schreuder T.C.M.A., Blokzijl H., Faber K.N. (2018). Disturbed Vitamin A Metabolism in Non-Alcoholic Fatty Liver Disease (NAFLD). Nutrients.

[26] Berry D.C., Noy N. (2012). Signalling by vitamin A and retinol-binding protein in regulation of insulin responses and lipid homeostasis. Biochim. Biophys. Acta Mol. Cell Biol. Lipids.

[27] Netto M.P., Priore S.E., Franceschini S.C.C. (2006). Indicators of the nutritional state of vitamin A. J. Braz. Soc. Food Nutr..

[28] Wortsman J., Matsuoka L.Y., Chen T.C., Lu Z., Holick M.F. (2000). Decreased bioavailability of vitamin D in obesity. Am. J. Clin. Nutr..

[29] Trasino S.E., Tang X.-H., Jessurun J., Gudas L.J. (2015). Obesity Leads to Tissue, but not Serum Vitamin A Deficiency. Sci. Rep..

[30] Coronel J., Pinos I., Amengual J. (2019). β-carotene in Obesity Research: Technical Considerations and Current Status of the Field. Nutrients.

[31] Miller A.P., Coronel J., Amengual J. (2020). The role of β-carotene and vitamin A in atherogenesis: Evidences from preclinical and clinical studies. Biochim. Biophys. Acta Mol. Cell Biol. Lipids.

[32] Mounien L., Tourniaire F., Landrier J.-F. (2019). Anti-Obesity Effect of Carotenoids: Direct Impact on Adipose Tissue and Adipose Tissue-Driven Indirect Effects. Nutrients.

[33] Harari A., Coster A.C.F., Jenkins A., Xu A., Greenfield J.R., Harats D., Shaish A., Bonet D.S. (2020). Obesity and Insulin Resistance Are Inversely Associated with Serum and Adipose Tissue Carotenoid Concentrations in Adults. J. Nutr..

[34] Brun P.J., Grijalva A., Rausch R., Watson E., Yuen J.J., Das B.C., Shudo K., Kagechika H., Leibel R.L., Blaner W.S. (2015). Retinoic acid receptor signaling is required to maintain glucose-stimulated insulin secretion and β-cell mass. FASEB J..

[35] Roohbakhsh A., Karimi G., Iranshahi M. (2017). Carotenoids in the treatment of diabetes mellitus and its complications: A mechanistic review. Biomed. Pharmacother..

[36] Östh M., Öst A., Kjolhede P., Stralfors P. (2014). The concentration of β-carotene in human adipocytes, but not the whole-body adipocyte stores, is reduced in obesity. PLoS ONE.

[37] Beydoun M.A., Chen X., Jha K., Beydoun H.A., Zonderman A.B., Canas J.A. (2019). Carotenoids, vitamin A, and their association with the metabolic syndrome: A systematic review and meta-analysis. Nutr. Rev..

[38] Zhou Y., Zhou J., Zhang Y., Tang J., Sun B., Xu W., Wang X., Chen Y., Sun Z. (2020). Changes in Intestinal Microbiota Are Associated with Islet Function in a Mouse Model of Dietary Vitamin A Deficiency. J. Diabetes Res..

[39] Godala M., Materek-Kuśmierkiewicz I., Moczulski D., Rutkowski M., Szatko F., Gaszyńska E., Tokarski S., Kowalski J. (2017). The risk of plasma vitamin A, C, E and D deficiency in patients with metabolic syndrome: A case-control study. Adv. Clin. Exp. Med..

[40] Kuang H., Wei C.H., Wang T., Eastep J., Li Y., Chen G. (2019). Vitamin A Status Affects Weight Gain and Hepatic Glucose Metabolism in Rats Fed a High-Fat Diet. Biochem. Cell Biol..

[41] Mody N. (2017). Alterations in vitamin A/retinoic acid homeostasis in diet-induced obesity and insulin resistance. Proc. Nutr. Soc..

[42] Dos Santos P.P., Andrade L.D.A., Flôres S.H., Rios A.D.O. (2018). Nanoencapsulation of carotenoids: A focus on different delivery systems and evaluation parameters. J. Food Sci. Technol..

[43] Dias M.I., Ferreira I.C.F.R., Barreiro M.F. (2015). Microencapsulation of bioactives for food applications. Food Funct..

[44] Suganya V., Anuradha V. (2017). Microencapsulation and Nanoencapsulation: A Review. Int. J. Pharm. Clin. Res..

[45] Melo R.L.F., Souza I.C.C., Carvalho A.J.R., Bezerra E.M., Costa R.F. (2020). Nanoparticles as biological tools: An exploratory review. Res. Soc. Dev..

[46] Hu Y., Zhang L., Zhang Y., Xiong H., Wang F., Wang Y., Lu Z. (2020). Effects of starch and gelatin encapsulated vitamin A on growth performance, immune status and antioxidant capacity in weaned piglets. Anim. Nutr..

[47] Rovoli M., Pappas I., Lalas S., Gortzi O., Kontopidis G. (2018). In vitro and in vivo assessment of vitamin A encapsulation in a liposome-protein delivery system. J. Liposome Res..

[48] Gonçalves A., Estevinho B.N., Rocha F. (2016). Microencapsulation of vitamin A: A review. Trends Food Sci. Technol..

[49] Azeredo H.M.C. (2005). Encapsulação: Aplicação à tecnologia de alimentos. Alim. Nutr..

[50] Sachaniya J., Savaliya R., Goyal R., Singh S. (2018). Liposomal formulation of vitamin A for the potential treatment of osteoporosis. Int. J. Nanomed..

[51] Liang R., Shoemaker C.F., Yang X., Zhong F., Huang Q. (2013). Stability and Bioaccessibility of β-Carotene in Nanoemulsions Stabilized by Modified Starches. J. Agric. Food Chem..

[52] Rocha F., Sugahara L.Y., Leimann F.V., Oliveira S., Brum E.D.S., Calhelha R.C., Barreiro M.F., Ferreira I.C.F.R., Ineu R.P., Gonçalves O.H. (2018). Nanodispersions of β-carotene: Effects on antioxidant enzymes and cytotoxic properties. Food Funct..

[53] Baek E.J., Garcia C.V., Shin G.H., Kim J.T. (2020). Improvement of thermal and UV-light stability of β-carotene-loaded nanoemulsions by water-soluble chitosan coating. Int. J. Biol. Macromol..

[54] Medeiros A.K.D.O.C., Gomes C.D.C., Amaral M.L.Q.D.A., De Medeiros L.D.G., Medeiros I., Porto D.L., Aragão C.F.S., Maciel B.L.L., Morais A.H.D.A., Passos T.S. (2019). Nanoencapsulation improved water solubility and color stability of carotenoids extracted from Cantaloupe melon (*Cucumis melo* L.). Food Chem..

[55] de Oliveira G.L.R., Medeiros I., Nascimento S.S.D.C., Viana R.L.S., Porto D.L., Rocha H.A.O., Aragão C.F.S., Maciel B.L.L., de Assis C.F., Morais A.H.D.A. (2021). Antioxidant stability enhancement of carotenoid rich-extract from Cantaloupe melon (*Cucumis melo* L.) nanoencapsulated in gelatin under different storage conditions. Food Chem..

[56] Liu G., Zhou Y., Chen L. (2019). Intestinal uptake of barley protein-based nanoparticles for β-carotene delivery. Acta Pharm. Sin. B.

[57] Liu X., Wanga P., Zoub Y.X., Luoa Z.G., Tamer T.M. (2020). Co-encapsulation of Vitamin C and β-Carotene in liposomes: Storage stability, antioxidant activity, and in vitro gastrointestinal digestion. Food Res. Int..

[58] Aditya N.P., Ko S. (2015). Solid lipid nanoparticles (SLNs): Delivery vehicles for food bioactives. RSC Adv..

[59] Salah E., Abouelfetou M.M., Pana Y., Chena D., Xie S. (2020). Solid lipid nanoparticles for enhanced oral absorption: A review. Colloids Surf. B Biointerfaces.

[60] Meng Q., Long P., Zhou J., Ho C.-T., Zou X., Chen B., Zhang L. (2019). Improved absorption of β-carotene by encapsulation in an oil-in-water nanoemulsion containing tea polyphenols in the aqueous phase. Food Res. Int..

[61] Luo X., Zhou Y., Bai L., Liu F., Deng Y., McClements D.J. (2017). Fabrication of β-carotene nanoemulsion-based delivery systems using dual-channel microfluidization: Physical and chemical stability. J. Colloid Interface Sci..

[62] Nakagawa K., Miyazawa T., Harigae T., Onuma R., Kimura F., Fujii T., Miyazawa T. (2015). Distribution of β-carotene-encapsulated polysorbate 80-coated poly(d, l-lactide-co-glycolide) nanoparticles in rodent tissues following intravenous administration. Int. J. Nanomed..

[63] Yao M., McClements D.J., Xiao H. (2015). Improving oral bioavailability of nutraceuticals by engineered nanoparticle-based delivery systems. Curr. Opin. Food Sci..

[64] Acosta E. (2009). Bioavailability of nanoparticles in nutrient and nutraceutical delivery. Curr. Opin. Colloid Interface Sci..

